# Electric Field Analysis of Breast Tumor Cells

**DOI:** 10.4061/2011/235926

**Published:** 2011-12-01

**Authors:** V. Gowri Sree, K. Udayakumar, R. Sundararajan

**Affiliations:** ^1^Anna University, Chennai 600 025, India; ^2^Purdue University, West Lafayette, IN 47907, USA

## Abstract

An attractive alternative treatment for malignant tumors that are refractive to conventional therapies, such as surgery, radiation, and chemotherapy, is electrical-pulse-mediated drug delivery. Electric field distribution of tissue/tumor is important for effective treatment of tissues. This paper deals with the electric field distribution study of a tissue model using MAXWELL 3D Simulator. Our results indicate that tumor tissue had lower electric field strength compared to normal cells, which makes them susceptible to electrical-pulse-mediated drug delivery. This difference could be due to the altered properties of tumor cells compared to normal cells, and our results corroborate this.

## 1. Introduction

 Electroporation, a nonthermal phenomenon, is used to enhance the permeability of biological cells and tissues [1–4]. Here, an attempt has been made to study the electric field's effect on tumor tissues thereby leading to the efficacy of electroporation. Electroporation involves the rapid structural rearrangement of membrane, in response to an electrically applied electric field. A noticeable effect is a rapid increase in electrical conductivity attributed to the formation of pores in the bilayer lipid membrane. Literatures have shown significant trend of progressive electrical changes according to the proliferative characteristics of breast epithelial cells. Physiologists also further postulated that malignant transformation resulted from sustained depolarization and a failure of the cell to repolarize after cell division, making the area where cancer develops relatively depolarized when compared to their nondividing or resting counterparts [5]. It leads to the rupturing of membrane wall which can be either reversible or irreversible. Electroporation generally depend upon the magnitude and the duration of the voltage, and the field applied. The membrane potential *V*_*m*_ is given as [6]

(1) $$V_{m}=1.5ER\cos\delta ,$$

where *R* is the cell radius and **δ** is the angle between the electric field *E* and the radius vector. A detailed study of electric field distribution is necessary for an effective understanding of the tissue's behavior when subjected to electric field. In this study, a 3D modeling of tumor and normal tissues are simulated using ANSOFT. The behavior of tissue and the effect of electric field on them are noted in each case. Literature has indicated that changes in the electrical properties of abnormal breast are more significant compared to the breast normal tissues [7]. The surface potentials are sensitive to the presence of tumour, location and placement of the electrodes [8]. The results can be used as a complement to experimental analysis, essential for effective manipulation of tissues for practical, real-life applications, such as electroporation-mediated gene therapy and enhancement of chemo-drug delivery (electrochemotherapy) [1–4].

## 2. Simulation of Tissue Model

For fast and accurate simulation results, we chose the Maxwell's FEM Software from ANSOFT Corporation, USA. For this simulation study, a slightly modified version of the more detailed electrical model of a tumor tissue reported by Surowiec et al. [9] was used.

**Figure 1:**
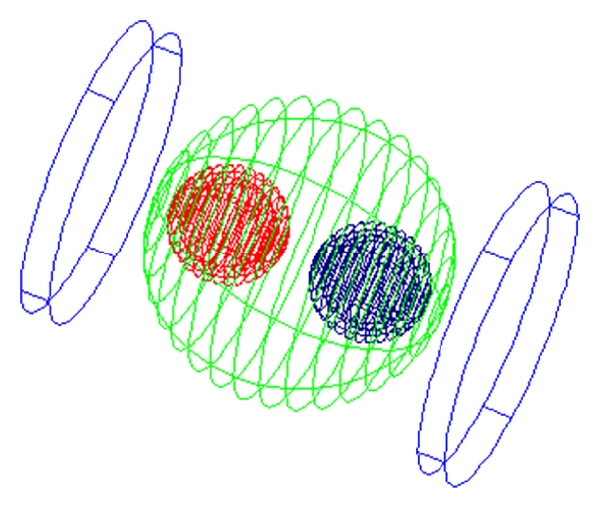
Tissue model with parallel plate electrodes.

 Three-dimensional models of breast lobe with single tumor and normal cell, with two tumor cells and two normal cells, are simulated for both needle and plate electrode configurations. The model developed using Maxwell 3D Simulator is shown in Figure 1. The tumor cell and normal cell are designed as spherical cells. The MAXWELL simulator is an interactive package that uses finite element analysis (FEA) to solve three-dimensional electrostatic problems. The normal and the tumor cell permittivity and conductivity parameters used for the model are shown in Table 1 [9, 10]. A high voltage of the order of 1200 V/cm is applied at 1 kHz frequency.

## 3. Results and Analysis

### 3.1. 3D Electric Field Analysis of Tissues

**Figure 2:**
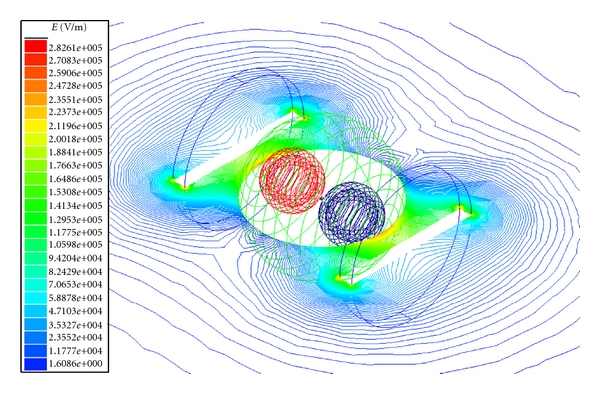
Electric field distribution for breast lobe tissue with normal cells (plane electrodes).

**Figure 3:**
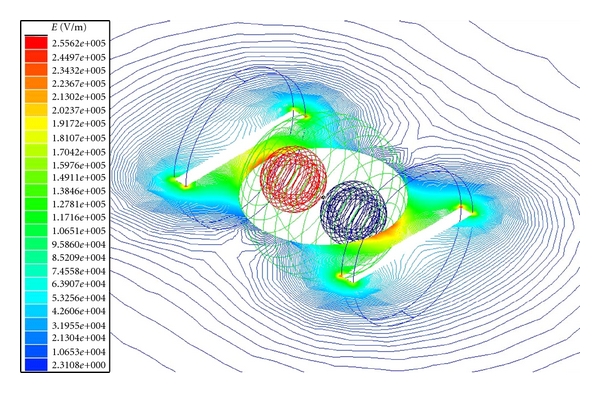
Electric field distribution for breast lobe tissue with tumor cells (plane electrodes).

**Figure 4:**
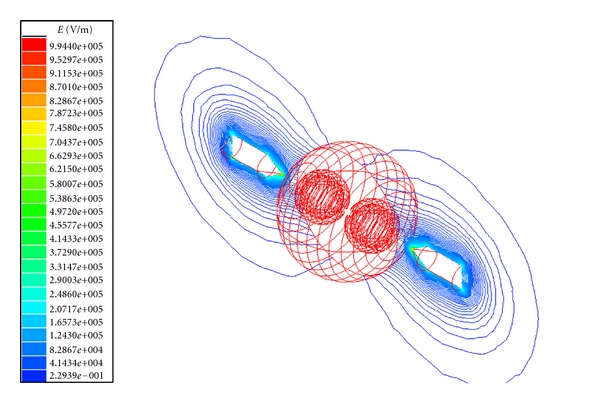
Electric field distribution for breast lobe tissue with tumor cells (needle electrodes).

The electric field across the tissue model for plate electrodes configuration is shown in Figures 2 and 3 for normal and tumor cell tissues. The electric field intensity is more for the normal cell tissue than the tumor cell tissue. This could be due to change in membrane structure, composition, and minerals in the tumor cell compared to the normal cell [11]. Similar results were obtained also for needle electrode configuration (Figure 4).

From this electric field distribution values, the transmembrane potential is calculated for the applied voltage. Also, from the 3D simulated model of different tissue model the capacitance value is noted and this value is applied to the electrical model using MATLAB.

### 3.2. Electrical Model of the Cell

**Figure 5:**
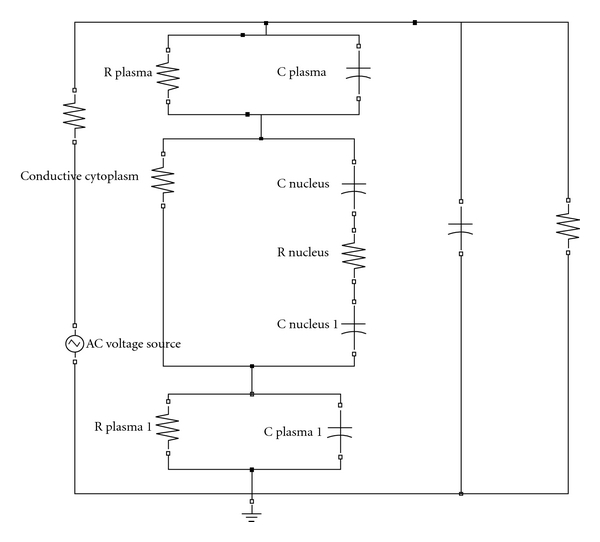
Electrical model of a cell with nucleus.

For this simulation study, a slightly modified version of the more detailed electrical model of a cell reported by Schoenbach's team [12, 13] is used.  Figure 5 illustrates the cell model used in this study. The rightmost R and C elements correspond to the medium. The entire breast lobe with tissues is assumed as a single cell, and simulation is done. Here, the tissue is modeled as a homogenous conductive medium (cytoplasm) surrounded by a leaky dielectric membrane.

In the above electrical model, the capacitance of the normal cell is replaced by the capacitance value obtained from the ANSOFT model of the cancer cell developed in this study. To this electrical model, a pulsed electric field of the order of 1200 V/cm is applied with a pulse width of 100 *μ*sec and for a time period of one second.

**Figure 6:**
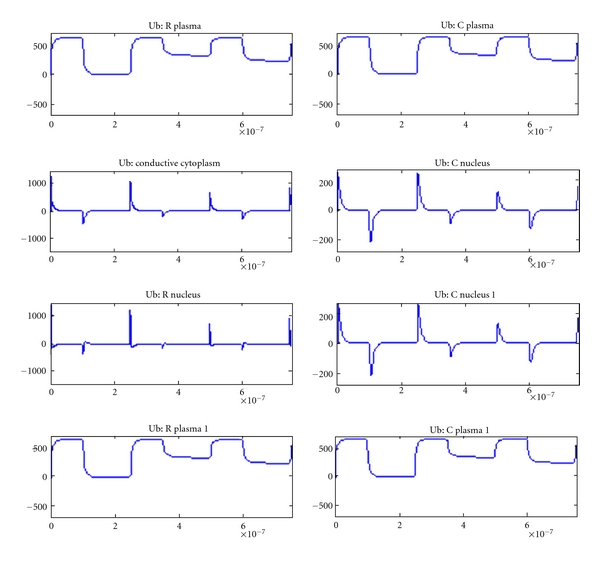
Voltage across various cell elements in tissue with normal and tumor cells.

**Figure 7:**
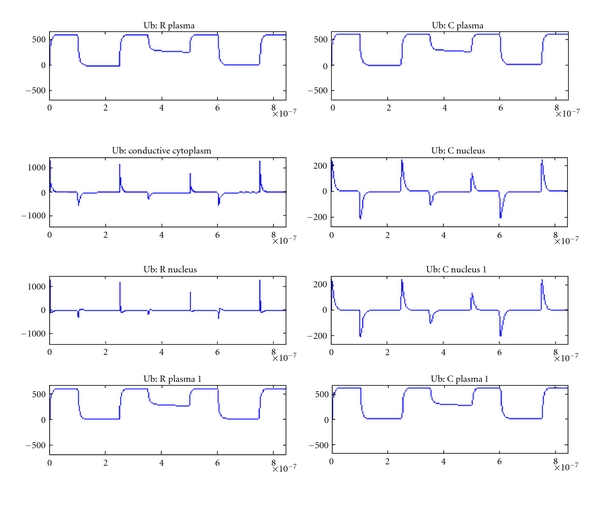
Voltage across various cell elements in tissue with two normal cells.

**Figure 8:**
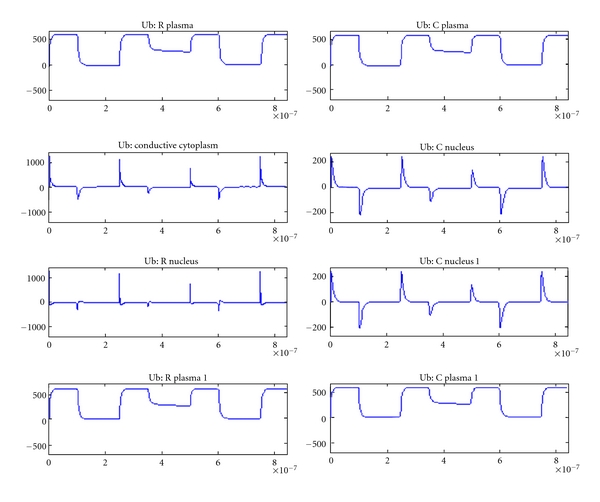
Voltage across various cell elements in tissue with two tumor cells.

**Figure 9:**
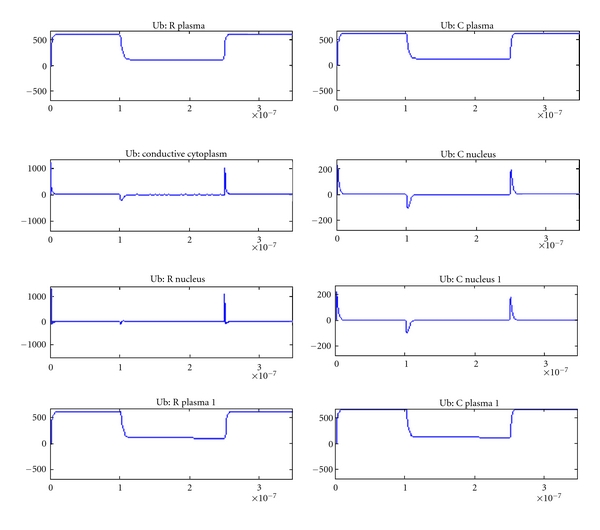
Voltage across various cell elements in tissue with tumor cells (needle electrode configuration).

**Figure 10:**
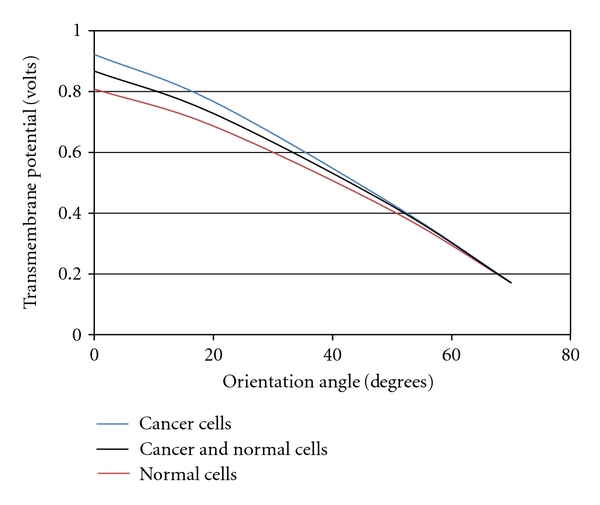
Variation of transmembrane potential with orientation.

The variation in voltage (*y*-axis) across each element with respect to time (*x*-axis) is shown in Figures 6–10 for various tissue models studied using plate electrode configuration.  Figure 6 shows the voltage across various cell elements, such as nucleus, plasma, and conductive cytoplasm for tissue with a normal cell and a tumor cell.

Figure 7 shows the voltages for a tissue with two normal cells and Figure 8 shows that of a tissue with two tumor cells (plate electrodes). There is slight variation in the tumor tissue model compared to normal tissue.  Figure 9 shows that for two tumor tissues using needle electrodes. There is noticeable difference between the voltages and their profiles for various elements between these two electrode configurations indicating the effect of electrode on the electroporation efficacy [14, 15].

### 3.3. Transmembrane Potential (TMP) and Its Comparison

Figure 10 shows the influence of the electric field orientation on the transmembrane potential (TMP). The orientation is varied from 0 to 70 degrees, and the TMP is evaluated for the applied voltage between the electrodes. It can be inferred that the transmembrane potential tends to decrease with increase in orientation. Also, the transmembrane potential of tumor tissue is less than that of the normal tissue. This could also be attributed to the altered cell membrane and other cell parameters of the tumor cell compared to the normal cell.

### 3.4. Nuclear Membrane Potential and Its Comparison

Table 2 shows the influence of the electric field orientation on the nuclear membrane potential (NMP). The orientation is varied from 0 to 70 degrees, and the NMP is evaluated for the applied voltage between the electrodes. It can be inferred that the NMP tends to decrease with increase in orientation. Also, the NMP of tumor tissue is less than that of the normal tissue. This could also be attributed to the altered cell membrane and other cell parameters of the tumor cell compared to the normal cell.

## 4. Discussion and Conclusions

Cancer cells have different electrical and metabolic properties due to abnormalities in structures [11]. A healthy cell membrane potential is strongly linked to the control of cell membrane transport and proliferation mechanisms as well as DNA activity, protein synthesis, and aerobic energy production. Since cancer cells cannot maintain a normal membrane potential, they will have electronic dysfunctions that will impede repair and the reestablishment of normal metabolic functions. Therefore, a key therapeutic method for cell repair and cancer treatment would be to reestablish a healthy membrane potential in the body's cells.

 In our research, the electric field distribution in normal and tumor tissues was investigated using 3D finite element analysis for plane and needle electrode configurations. The tumor tissues shows lower intensities of electric field compared to the normal tissues, possibly due to the altered characteristics of membrane potential, its composition and minerals such as potassium, magnesium, sodium, and calcium [11]. These results demonstrate the susceptibility of malignant cells to the electric field application and the relative robustness of the normal cells, illustrating the enhanced efficacy of the electrochemotherapy using lower drug doses. The field analysis results can be used for assessing effective treatment parameters of tumor tissues.

 The electrical characteristics of the membrane and the cytoplasm, such as the conductivity and permittivity of the membrane and the cytoplasm, as well as membrane thickness also govern the response due to electroporation in addition to the intensity and distribution of the electric field applied. Our results indicate that electric field analyses could be used for selecting suitable parameters for effective treatment of tumor tissues, since these parameters need to be optimized for various tumors/tissues and cells.

## Figures and Tables

**Table 1:** Electrical parameters used [9, 10].

Cell classification	Dielectric constant	Conductivity, S/cm
Normal cell I	3850	0.62
Normal cell II	2310	0.60
Cancer cell I	7052	0.26
Cancer cell II	5110	0.20
Medium	2000	0.20

**Table 2:** Nuclear membrane potential of tumor and normal tissues.

Degree	Cancer cells	Normal cells	Cancer & normal cells
0	0.9216	0.808	0.8671
20	0.7669	0.6863	0.7278
50	0.4293	0.407	0.4234
70	0.1709	0.17014	0.1698
